# Synthesis of a Conformationally Stable Atropisomeric Pair of Biphenyl Scaffold Containing Additional Stereogenic Centers

**DOI:** 10.3390/molecules24030643

**Published:** 2019-02-12

**Authors:** Chi-Tung Yeung, Wesley Ting Kwok Chan, Wai-Sum Lo, Ga-Lai Law, Wing-Tak Wong

**Affiliations:** 1 The Hong Kong Polytechnic University Shenzhen Research Institute, Shenzhen 518000, PR China; chitung.yeung@polyu.edu.hk (C.-T.Y.); wai-sum.lo@polyu.edu.hk (W.-S.L.); ga-lai.law@polyu.edu.hk (G.-L.L.); 2 State Key Laboratory of Chemical Biology and Drug Discovery, Department of Applied Biology and Chemical Technology, The Hong Kong Polytechnic University, Hung Hom, Kowloon 999077, Hong Kong; wesley.chan@polyu.edu.hk

**Keywords:** atropisomer, asymmetry, hydrogen bond, *N*-nitroso aldol reaction

## Abstract

The synthesis of a new CF_3_-containing stereogenic atropisomeric pair of ortho-disubstituted biphenyl scaffold is presented. The atropisomers are surprisingly conformationally stable for isolation. X-ray structures show that their stability comes from an intramolecular hydrogen bond formation from their two hydroxyl groups and renders the spatial arrangement of their peripheral CF_3_ and CH_3_ groups very different. The synthesized stereogenic scaffold proved to be effective in catalyzing the asymmetric *N*-nitroso aldol reaction of enamine and nitrosobenzene. Compared to similar scaffolds without CF_3_ groups, one of our atropisomer exhibits an increase in enantioselectivity in this reaction.

## 1. Introduction

The nitroso aldol reaction is one of the most powerful methods to introduce nitrogen and oxygen moieties at the α-position of an enolizable carbonyl compound to give synthetically useful *N*/*O*-substituted carbonyl intermediates for the synthesis of a variety of natural products and pharmaceutical compounds [1,2,3,4,5]; however, high reactivity of both the nitrogen and oxygen atoms in a nitroso compound towards the nucleophile has made it difficult to control *N*- or *O*-regioselective additions to enolate. It is generally understood that a judicious choice of a suitable promoter (hydrogen bond activator or Lewis acid, etc.) and enolate (silyl- or lithiate-) are determinant factors to control this regioselectivity to either hydroxyamino or aminooxy compounds [6,7,8].

The past two decades have seen great progress in the research of asymmetric organocatalytic *O*-nitroso aldol reactions [9,10,11,12,13,14,15,16]; in contrast, research in the *N*-nitroso aldol reaction is rather limited [17,18,19,20,21]. Most of the *N*-nitroso aldol stereogenic catalysts contain a 2° amino unit for the in situ generation of enamine prior to reacting with the nitroso compound. Another catalyst design for this reaction is to consider activating the nitroso compound and hence react with a preformed enamine of interest to selectively form the hydroxyamino product. Yamamoto and his co-workers reported a catalytic *N*-nitroso aldol reaction system based on a hydrogen bond forming di-alcoholic TADDOL to give the hydroxyamino products from cyclohexene enamine in good yields and enantioselectivities [22]. To the best of our knowledge, this is the only report on organocatalytic *N*-nitroso aldol reactions with a preformed enamine with good % ee; as such, there is still much room for improvement and investigation.

In the literature, catalysts with ortho-tetrasubstituted biaryl backbones, such as binaphthyl, are well-known efficient catalysts for many asymmetric reactions [23,24,25,26]. For catalysts with ortho-disubstituted biphenyl backbones, research in this field is rather limited. The reason may be attributed to uncontrollable internal rotation at the biphenyl backbone, which leads to a co-existence of the atropisomers, *R*_a_ and *S*_a_, and this is generally believed to adversely affect the enantioselectivity in catalytic reactions. Although limited, some catalysts based on ortho-disubstituted biphenyl backbones are occasionally reported to give high enantioselectivities in organocatalysis [27,28]. There is great potential to develop efficient organocatalysts based on an ortho-disubstituted biphenyl backbone if the corresponding rotation at the biphenyl axis can be well-regulated by steric effect or other delicate interactions.

**Scheme 1:**
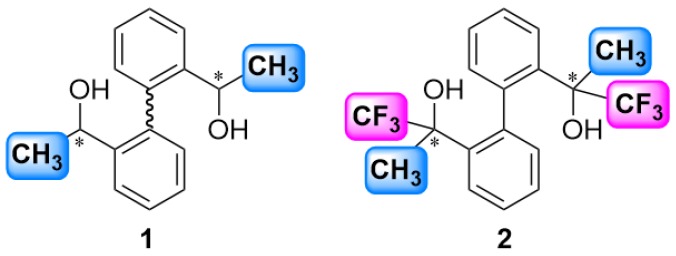
Stereogenic biphenyl scaffold-based di-alcoholic compounds.

As part of our research program on studying supramolecular formation behavior with some stereogenic biphenyl di-alcoholic compounds, we discovered that a series of strong intermolecular hydrogen bonding interactions are the major force in helping to maintain a high atropisomeric ratio (ratio of *R*_a_ to *S*_a_) of a stereolabile compound **1** (Scheme 1) [29]. Pure atropisomer **1** can be isolated but a deterioration in atropisomeric ratio (9:1 to 7:3) was observed after dissolution in organic solvents. Inspired by Kumadaki and his co-workers’ pioneering studies on the development of axially dissymmetric ligands with enhanced hydroxyl acidity by incorporating different perfluoroalkyl groups [30,31], we would like to report our new findings: the synthesis of a new axially stereogenic biphenyl scaffold-based di-alcoholic compound **2** bearing two additional CF_3_ substituents. A surprise formation of a pair of stable and isolated atropisomers (*R*_a_ and *S*_a_) with their axial chirality being fixed and stabilized by intramolecular hydrogen bonds (OH···OH) (O–H···O) was observed. The formation of an atropisomeric pair with a substantially less bulky biphenyl diol was also reported by Kumadaki and his co-workers [32]; however, the pair was susceptible to atropisomerization, leading to the major formation of one of their isomers.

In our study, **1** and **2** were preliminarily screened as organocatalysts in an asymmetric *N*-nitroso aldol reaction of enamine with nitrosobenzene.

## 2. Results and Discussion

**Scheme 2:**
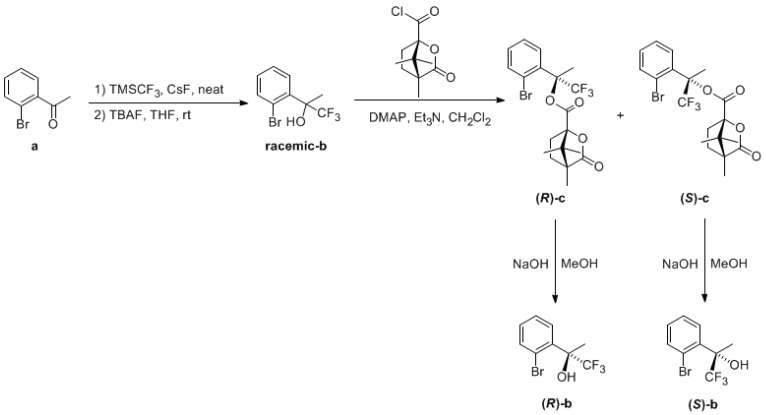
Synthetic pathway for enantiopure **(*R*)-b** or **(*S*)-b**.

**Figure 1:**
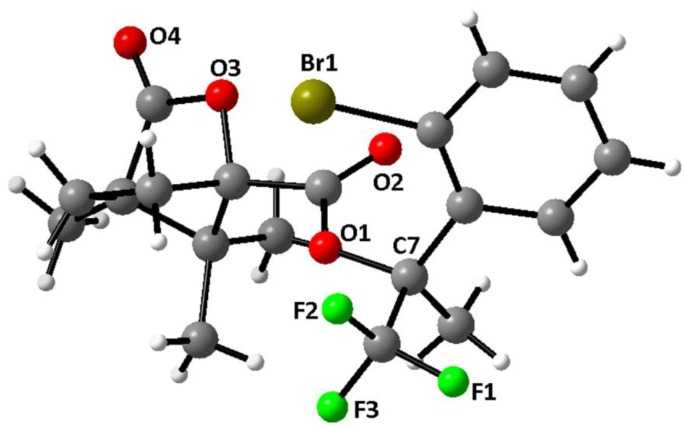
X-ray crystal structure of **(*S*)-c**.

Our approach to the synthesis of a key intermediate, enantiopure 2-(2-bromophenyl)-1,1,1-trifloropropan-2-ol **b**, is presented in Scheme 2. Firstly, the CF_3_ substituent was introduced to the carbonyl group of 2′-bromoacetophenone **a** by trifluoromethylation with trifluoromethyltrimethylsilane. After removing the trimethylsilyl group with tetrabutylammonium fluoride, **b** was obtained as a racemic mixture with a good yield (85%). Enantiopure **b** can be obtained by chiral resolution of **racemic-b** with (1*S*)-(─)-camphanic chloride. The corresponding diastereomers, **(*R*)-c** or **(*S*)-c**, can be separately easily with column chromatography, and their diastereoselectivities are high enough without the need for further purification with recrystallization. For **(*S*)-b**, its absolute configuration was revealed to be (*S*) at C-7 with an X-ray crystal structure (Figure 1). After removing the camphanic substituent, enantioselectivity of the corresponding **(*R*)-b** or **(*S*)-b** was checked with HPLC and found to be higher than 99% (Figure S1).

**Scheme 3:**
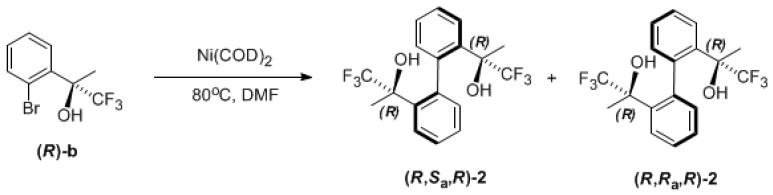
Homocoupling reaction of enantiopure **b** with Ni(COD)_2_.

**Figure 2:**
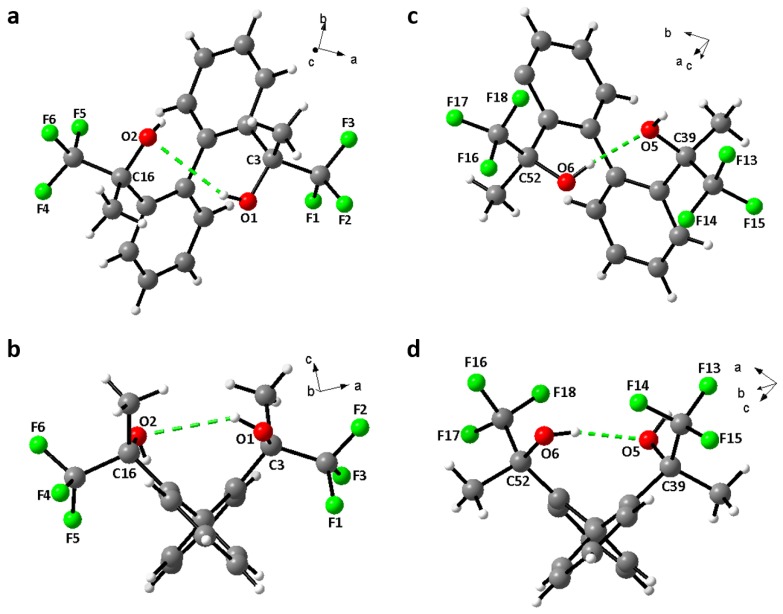
X-ray crystal structures of **(*R***,***S*_a_**,***R*)-2** (a, b) and **(*R***,***R*_a_**,***R*)-2** (c, d) at two different orientations to show (i) ***S*_a_** or ***R*_a_** atropselectivities; (ii) relative different spatial arrangements of the CF_3_- and CH_3_-substituents. Hydrogen bond interactions are highlighted as green dashed lines. Torsion angle of biphenyl rings for **(*R***,***S*_a_**,***R*)-2** is 90.4° whereas for **(*R***,***R*_a_**,***R*)-2** it is 85.3°.

Next, the enantiopure **(*R*)-b** was utilized for a homocoupling reaction with Ni(COD)_2_ for the synthesis of compound **2**. In general, protection of the alcohol moiety with acetyl chloride or chloromethyl methyl ether was required prior to homocoupling [33]; however, we found that a reasonable yield (66%) of the coupling products could also be obtained without the need of protection. Interestingly, two coupled products of **2** with a ratio of 1:1.2 were observed by analysis of the crude reaction mixture with ^1^H NMR, and the two products were then separated with column chromatography (Scheme 3.). Fortunately, single crystals suitable for diffraction studies were obtained from these two coupled products by evaporation from diethyl ether and they were confirmed to be the two atropisomers, **(*R***,***S*_a_**,***R*)-2** and **(*R***,***R*_a_**,***R*)-2** (Figure 2).

For both structures, no extensive intermolecular O–H···O hydrogen bond interactions were observed for formation of supramolecular structures; instead, only intramolecular hydrogen bonds were found. This is in contrast to our previous findings in the formation of supramolecular helical structures with the aid of extensive intermolecular hydrogen bonds of **1**. In addition, the C–O distances in the current two atropisomers (**(*R***,***S*_a_**,***R*)-2**: C3–O1 (1.409(3) Å) and C16–O2 (1.417(3) Å); **(*R***,***R*_a_**,***R*)-2**: C39–O5 (1.419(5) Å) and C52–O6 (1.420(4) Å)) were found to be shorter than those of **1 [29]** (1.43 Å). Short C–O distances for other CF_3_-containing compounds was observed in the literature [33].

In the two structures, **(*R***,***R*_a_**,***R*)-2** exhibited a stronger hydrogen bond (O–H···O) interaction than **(*R***,***S*_a_**,***R*)-2** as demonstrated in **(*R***,***R*_a_**,***R*)-2** having a shorter and more linear hydrogen bond (D(O6–H···O5): 1.920(4) Å; ∠(O6–H∙∙∙O5): 164.53(12)°) than that of **(*R***,***S*_a_**,***R*)-2** (D(O1–H···O2): 2.646(2) Å; ∠(O1–H∙∙∙O2): 137.56(14)°). Moreover, the relative spatial arrangement of the larger size CF_3_- and smaller size CH_3_-groups for them was different. For **(*R***,***S*_a_**,***R*)-2**, the CF_3_ groups were located at the side far from the intramolecular hydrogen bond unit whereas for **(*R***,***R*_a_**,***R*)-2**, the CF_3_ group was in a closer position. The difference in their spatial arrangement may suggest different steric environments around their respective hydrogen bond units.

In solution state, both of the atropisomers were stable. The circular dichroism (CD) spectra of **(*S***,***R*_a_**,***S*)-2** or **(*S***,***S*_a_**,***S*)-2** showed two exciton coupling bands in CHCl_3_. The corresponding bands were found to attenuate with 32% and 17% in MeOH (Figure S2). Compared to **1**, its extent of signal attenuations from CHCl_3_ to MeOH was not as great (80% for **1)** [29]. Solution stability of **(*S***,***R*_a_**,***S*)-2** and **(*S***,***S*_a_**,***S*)-2** could also be directly investigated by dissolving them in d-solvents (such as d-chloroform, d-methanol, d-benzene, d-dimethyl sulfoxide, and d-acetic acid) of different polarities. No atropisomerization from **(*S***,***R*_a_**,***S*)-2** to **(*S***,***S*_a_**,***S*)-2** or vice versa could be observed, even with heating of the corresponding atropisomers at 60 °C for 2 days. This was in contrast to **1,** which exhibited atropisomerization to give a 7:3 atropisomeric ratio in polar solvents in 1 day at room temperature [29], and this may indicate that the intramolecular hydrogen bonds in **(*S***,***R*_a_**,***S*)-2** and **(*S***,***S*_a_**,***S*)-2** were strong enough to keep their conformation intact, as the flipping of biphenyl backbones from one atropisomer to another atropisomer may not be preferable.

With the compounds **1**, **(*S***,***R*_a_**,***S*)-2** and **(*S***,***S*_a_**,***S*)-2** in hand, we firstly investigate the *N*-nitroso aldol reaction of morpholine enamine with nitrosobenzene at −50 °C (Table 1). Three of them were active catalysts for this reaction and resulted in 23 to 63% yields of the catalytic product hydroxylamine (entries 1 to 3). **(*S***,***R*_a_**,***S*)-2** gave a lower yield (38%) compared to the non-CF_3_-containing compound **1** (63%) but it is worthy to note that it exhibited a significantly higher enantioselectivity (44% ee) over compound **1** (12% ee) and, more importantly, its atropisomer **(*S***,***S*_a_**,***S*)-2** (8% ee). The absolute configurations of the hydroxylamines were the same as (*S*) and no undesired aminoxylated product was observed by TLC. Apart from morpholine enamine, other enamines were also tried. **(*S***,***R*_a_**,***S*)-2** could catalyze piperidine enamine but resulted in poor reactivity and only moderate enantioselectivity (entry 4). For the pyrrolidine enamine, interestingly, no desired product was obtained (entry 5). Apart from toluene, dichloromethane, diethyl ether, and tetrahydrofuran were also used in the reaction using **(*S***,***R*_a_**,***S*)-2** as catalyst and all gave higher yields (entries 6–8) than toluene (entry 2); however, the % ee achieved by toluene remained the highest among them. Furthermore, in toluene, both the yield and the enantioselectivity were further improved to 64% and 50% ee, respectively, when the reaction temperature dropped to −80 °C (entry 9). The yields and enantioselectivities were found to decrease when the catalyst loadings were lowered to 20 mol% (entry 10) or 10 mol% (entry 11). The enantiomeric pairs **(*S***,***R*_a_**,***S*)-2** and **(*S***,***S*_a_**,***S*)-2** were also investigated: ***(R***,***S*_a_**,***R*)-2** resulted in similar yield and % ee than its enantiomer **(*S***,***R*_a_**,***S*)-2** (entries 12 vs 9). Similar to **(*S***,***S*_a_**,***S*)-2** (entry 3), ***(R***,***R*_a_**,***R*)-2** (entry 13) gave a lower reactivity and enantioselectivity than its atropisomeric counterpart ***(R***,***S*_a_**,***R*)-2** (entry 12). The hydroxylamines obtained from ***(R***,***S*_a_**,***R*)-2** and ***(R***,***R*_a_**,***R*)-2** were in (*R*)-configuration.

## 3. Materials and Methods 

### 3.1. General Experimental Methods

The compound (1*R*,1′*R*)-1,1′-(biphenyl-2,2′-diyl)diethanol (**1**) was prepared according to our previous report [29]. All reagents were purchased commercially and used as received. Dichloromethane was dried over calcium hydride. Toluene, diethyl ether, and tetrahydrofuran were dried over 3Å molecular sieves. Anhydrous dimethylformamide (DMF) was available commercially. All other solvents were used without drying. Unless otherwise stated, all manipulations were carried out under nitrogen using the Schlenk line technique. NMR spectra were recorded on a Bruker Ultrashield Advance Pro 400 MHz instrument (Billerica, MA, USA). Chemical shifts of ^1^H and ^13^C were referenced internally to tetramethylsilane or solvent residue in d-solvent in parts per million (ppm). Chemical shifts of ^19^F were referenced externally with trifluoroacetic acid (−76.55 ppm). The absolute configuration was determined with X-ray crystallography. Mass spectra were obtained with an Agilent 7890A GC-Waters GCT Premier EI-TOF-MS (Santa Clara, CA, USA) or an Agilent 6540 ESI-QTOF-MS (Santa Clara, CA, USA). CD spectra were recorded with a Jasco J-801 spectropolarimeter with a 1 mm cell at 25 °C and presented as Δɛ in M^−1^cm^−1^.

### 3.2. X-ray Crystallography

The crystal data reported in the manuscript were collected on a Bruker D8-Venture system. Structures **(*R****,**S*****_a_***,**R*****)-2** and **(*R****,**R*****_a_***,**R*****)-2** were collected with Cu-Kα radiation while Mo-Kα was used for **(*S*)-c**. All data were collected at room temperature. Multi-scan absorption correction was applied by the SADABS program [34], and the SAINT program, Bruker-AXS 2014 APEX3 software suite (Madison, Wisconsin, USA), utilized for the integration of the diffraction. All structures were solved by direct method and were refined by a full-matrix least-squares treatment on *F^2^* using the SHELXLE program system [35]. The crystallographic data for the structural analyses have been deposited with the Cambridge Crystallographic Data Centre, and the data can be obtained free of charge via www.ccdc.cam.ac.uk/data_request/cif. CCDC No. 1,866,713 for **(*S*)-c**; 1,866,714 for **(*R***,***S*_a_**,***R*)-2;** and 1,866,718 for **(*R***,***R*_a_**,***R*)-2**.

### 3.3. Procedure for Synthesis of (R,S_a_,R)-2 and (R, R_a_,R)-2

#### 3.3.1. Synthesis of Racemic 2-(2-bromophenyl)-1,1,1-trifluoropropan-2-ol, racemic-b

Cesium fluoride (0.03 g, 0.20 mmol) was added to a solution of 2′-bromoacetophenone, a, (4 g, 20 mmol) and (trifluoromethyl)trimethylsilane (4.5 mL, 30.46 mmol) at 0 °C with stirring. Effervescence was observed. After no bubbles appeared, the reaction mixture was stirred at room temperature for 16 h. After rotary evaporating the excess reagent, tetrahydrofuran (10 mL) and then tetrabutylammonium fluoride (70%, 16 mL) were added subsequently to the residue. After stirring the reaction mixture at room temperature for 1 h, the mixture was diluted with water (50 mL), and the product was extracted with diethyl ether (30 mL × 3). The extract was dried with anhydrous magnesium sulfate and purified with column chromatography (petroleum ether/ethyl acetate, 20:1) to yield 2-(2-bromophenyl)-1,1,1-trifluoropropan-2-ol, racemic-b, (4.56 g, 17.0 mmol, 85% yield). ^1^H NMR was conducted (400 MHz, CDCl_3_, δ): 1.92 (s, 3H), 4.17 (s, 1H), 7.19 (t, *J* = 8 Hz, 1H), 7.33 (t, *J* = 8 Hz, 1H), 7.61 (t, *J* = 8 Hz, 2H). ^13^C NMR (100 MHz, CDCl_3_, one peak was missing due to overlapping, δ): 23.26, 76.78 (q, *J* = 29 Hz), 120.62, 125.59 (q, *J* = 286 Hz), 127.53, 130.18, 135.79, 136.22. ^13^C NMR (100 MHz, CD_3_OD, δ): 23.32, 76.61, (q, *J* = 29 Hz), 122.19, 127.36 (q, *J* = 284 Hz), 128.14, 130.92, 131.19, 137.04, 139.40. ^19^F NMR (376 MHz, CDCl_3_, δ): −77.75 (s). MS (EI): m/z = 267.97 (M^+^), 249.97 (M^+^ − H_2_O), 198.98 (base peak, M^+^ − CF_3_).

#### 3.3.2. Synthesis of ((1*S*,4*R*)-((*R*)-2-(2-bromophenyl)-1,1,1-trifluoropropan-2-yl) 4,7,7-trimethyl-3-oxo-2-oxabicyclo (2.2.1)heptane-1-carboxylate), (*R*)-c, and ((1*S*,4*R*)-((*S*)-2-(2-bromophenyl)-1,1,1-trifluoropropan-2-yl) 4,7,7-trimethyl-3-oxo-2-oxabicyclo (2.2.1)heptane-1-carboxylate), (*R*)-c

The compound (1*S*)-Camphanic chloride (10.02 g, 46.27 mmol) was added to a solution of 2-(2-bromophenyl)-1,1,1-trifluoropeopan-2-ol, **racemic-b**, (3.76 g, 14.00 mmol), triethylamine (6.45 mL, 46.27 mmol), and 4-(dimethylamino)pyridine (1.71 g, 14 mmol) in anhydrous dichloromethane (250 mL) at 0 °C under nitrogen. After stirring the reaction mixture at room temperature for 16 h, the mixture was washed with water, saturated sodium bicarbonate, and then brine (50 mL of each). The organic layer was then dried with magnesium sulfate, filtered, and concentrated. The crude residue was purified with column chromatography (petroleum ether/dichloromethane, 10:1). The **(*R*)-c**, ((1*S*,4*R*)-((*R*)-2-(2-bromophenyl)-1,1,1-trifluoropropan-2-yl) 4,7,7-trimethyl-3-oxo-2-oxabicyclo(2.2.1)heptane-1-carboxylate), eluted faster than the **(*S*)-c** ((1*S*,4*R*)-((*S*)-2-(2-bromophenyl)-1,1,1-trifluoropropan-2-yl) 4,7,7-trimethyl-3-oxo-2-oxabicyclo(2.2.1)heptane-1-carboxylate). **(*R*)-c** was (2.17 g, 4.99 mmol, 36% yield). The following procedures were conducted: ^1^H NMR (400 MHz, CDCl_3_, δ): 1.05 (s, 6H), 1.14 (s, 3H), 1.68–1.74 (m, 1H), 1.88–1.96 (m, 1H), 2.14–2.20 (m, 1H), 2.22 (s, 3H), 2.41–2.47 (m, 1H), 7.22 (t, *J* = 7.2 Hz, 1H), 7.37 (t, *J* = 7.2 Hz, 1H), 7.47 (d, *J* = 8Hz, 1H), 7.65 (d, *J* = 7.6Hz, 1H); ^13^C NMR (100 MHz, CDCl_3_, δ): 9.68, 16.53, 16.77, 21.62, 28.81, 30.99, 54.66, 54.97, 83.35 (d, *J* = 30 Hz), 90.55, 120.73, 123.92 (q, *J* = 283 Hz), 127.47, 129.73, 130.48, 132.87, 136.28, 164.99, 178.14; ^19^F NMR (376 MHz, CDCl_3_, δ): −77.07 (s); MS (ESI): calc. for C_19_H_20_BrF_3_O_4_Na (M + Na^+^): 471.0389, found 471.0380; **(*S*)-c** was (1.62 g, 3.72 mmol, 27% yield); ^1^H NMR (400 MHz, CDCl_3_, δ): 1.01 (s, 3H), 1.13 (s, 6H), 1.73–1.77 (m, 1H), 1.93–1.97 (m, 1H), 2.18–2.25 (m, 1H), 2.20 (s, 3H), 2.57–2.61 (m, 1H), 7.22 (t, *J* = 7.6 Hz, 1H), 7.36 (t, *J* = 7.6 Hz, 1H), 7.45 (d, *J* = 8Hz, 1H), 7.65 (d, *J* = 8 Hz, 1H); ^13^C NMR (CDCl_3_, δ): 9.64, 16.72, 16.86, 21.85, 29.02, 31.07, 54.40, 54.81, 83.29 (q, *J* = 30 Hz), 90.73, 120.63, 124.10 (q, *J* = 283 Hz), 127.53, 129.59, 130.53, 132.83, 136.29, 164.08, 177.73; ^19^F NMR (376 MHz, CDCl_3_, δ): −76.71 (s); MS (ESI): calc. for C_19_H_20_BrF_3_O_4_Na (M + Na^+^): 471.0389, found 471.0382. Crystals of the **(*S*)-c** were formed by slow evaporation of the product from diethyl ether solution. Crystals were good enough for X-ray crystal crystallography.

#### 3.3.3. Synthesis of (*R*)-2-(2-bromophenyl)-1,1,1-trifluoropropan-2-ol, (*R*)-b

Sodium hydroxide (0.46 g, 11.5 mmol) was added to a solution of **(*R*)-c**, (2.5 g, 5.8 mmol) in tetrahydrofuran (63 mL) and methanol (25 mL). After stirring the reaction mixture for 3 h, the solvent was removed. The residue was then purified with column chromatography (petroleum ether/ethyl acetate, 30:1) to yield (*R*)-2-(2-bromophenyl)-1,1,1-trifluoropropan-2-ol, **(*R*)-b**, (1.41 g, 5.26 mmol, 92% yield). The following were conducted: ^1^H NMR (400 MHz, CDCl_3_, δ): 1.93 (s, 3H), 4.15 (s, 1H), 7.21 (t, *J* = 8 Hz, 1H), 7.35 (t, *J* = 8 Hz, 1H), 7.61 (d, *J* = 8 Hz, 1H), 7.64 (d, *J* = 8 Hz, 1H); ^13^C NMR (100 MHz, CDCl_3_, one peak is missing due to overlapping, δ): 23.29, 76.80 (q, *J* = 30 Hz), 120.61, 125.59 (q, *J* = 285 Hz), 127.54, 130.20, 135.79, 136.20; ^13^C NMR (100 MHz, CD_3_OD, δ): 23.35, 76.64, (q, *J* = 29 Hz), 122.22, 127.38 (q, *J* = 285 Hz), 128.16, 130.94, 131.21, 137.06, 139.40; ^19^F NMR (376 MHz, CDCl_3_, δ): −77.74 (s); MS (EI): m/z = 267.97 (M^+^), 249.96 (M^+^ − H_2_O), 198.98 (base peak, M^+^ − CF_3_). The enantiomeric purity was determined with HPLC with AD-H column (Hexane/*i*-propanol: 98:2; flow rate: 0.5 mL/min) and compared with a racemic mixture according to the elution orders with retention times, t_S_ = 21.90 min and t_R_ = 25.55 min) to be >99% ee. The corresponding **(*S*)-b** was obtained with the same method from **(*S*)-c**.

#### 3.3.4. Synthesis of (*S*_a_)-(2*R*,2′′*R*)-((1,1′-biphenyl)-2,2′-diyl)bis(1,1,1-trifluoropropan-2-ol) (*R*,*S*a,*R*)-2 and (*R*_a_)-(2*R*,2′*R*)-((1,1′-biphenyl)-2,2′-diyl)bis(1,1,1-trifluoropropan-2-ol) (*R*,*R*a,*R*)-2

(*R*)-2-(2-bromophenyl)-1,1,1-trifluoropropan-2-ol, (*R*)-b, (2.98 g, 11.12 mmol) was added to a suspension of bis(1,5-cyclooctadiene)nickel(0) (1.38g, 5.00 mmol) in 10.0 mL anhydrous dimethylformamide under nitrogen. The mixture was heated at 80 °C for 16 h. After cooling to room temperature, the reaction was quenched by addition of 5% aqueous hydrochloric acid and then extracted with diethyl ether. The extract was dried with anhydrous magnesium sulfate and purified with column chromatography (petroleum ether/ethyl acetate, 20:1) to yield (*R*,*S*_a_,*R*)-2 (0.75 g, 1.99 mmol, 36%) and (*R*,*R*_a_,*R*)-2 (0.63 g, 1.67 mmol, 30%). The starting material, (*R*)-b (0.53 g, 1.97 mmol, 18%), was recovered. For (*R*,*S*_a_,*R*)-2, the following was conducted: ^1^H NMR (400 MHz, CDCl_3_, δ): 1.63 (s, 6H), 3.55 (s, 2H), 7.04 (d, *J* = 8 Hz, 2H), 7.27 (t, *J* = 8 Hz, 2H), 7.34 (t, *J* = 8 Hz, 2H), 7.60 (d, *J* = 8 Hz, 2H); ^13^C NMR (100 MHz, CDCl_3_, δ): 25.46, 77.67 (q, *J* = 28 Hz), 125.75 (q, *J* = 5 Hz), 125.96 (q, *J* = 285 Hz), 126.94, 127.14, 131.96, 135.67, 141.76; ^19^F NMR (376 MHz, CDCl_3_, δ): −75.91 (s); MS (ESI): calc. for C_18_H_16_F_6_O_2_Na (M + Na^+^): 401.0947, found 401.0942. For (*R*,*R*_a_,*R*)-2, the following was conducted: ^1^H NMR (400 MHz, CDCl_3_, δ): 1.79 (s, 6H), 2.74 (s, 2H), 7.08 (d, *J* = 8 Hz, 2H), 7.34 (t, *J* = 8 Hz, 2H), 7.40 (t, *J* = 8 Hz, 2H), 7.45 (d, *J* = 8 Hz, 2H); ^13^C NMR (100 MHz, CDCl_3_, one peak is missing due to overlapping, δ): 25.14, 77.63 (q, *J* = 30 Hz), 125.32 (q, *J* = 284 Hz), 127.77, 128.69, 132.01, 135.44, 140.25; ^13^C NMR (100 MHz, CD_3_OD, δ): 25.98, 77.76 (q, *J* = 29 Hz), 127.15 (q, *J* = 284 Hz), 127.30, 127.80, 128.86, 133.74, 136.16, 144.77; ^19^F NMR (376 MHz, CDCl_3_, δ): −79.16 (s); MS (ESI): calc. for C_18_H_16_F_6_O_2_Na (M + Na^+^): 401.0947, found 401.0939. Crystals of (*R*,*S*_a_,*R*)-2 or (*R*,*R*_a_,*R*)-2 were formed by slow evaporation of the corresponding products from diethyl ether solutions, respectively. The quality of the crystals was good enough for X-ray crystal crystallography.

### 3.4. General Procedure for Asymmetric N-Nitroso Aldol Reaction

To a two-necked pear shape flask charged with nitrosobenzene (17.9 mg, 0.167 mmol) and compound **1** or **2** (0.05 mmol) was added anhydrous toluene (0.67 mL). The reaction mixture was stirred at room temperature under nitrogen for 30 min. After cooling the reaction mixture at a desired temperature (−50 °C or −80 °C), corresponding enamine (0.167 mmol) in anhydrous toluene (0.33 mL) was added over 1 h and stirred at the same temperature for 1 day (−50 °C) or 2 days (−80 °C). The reaction mixture was quenched with saturated brine (6 mL) and the aqueous layer was extracted with dichloromethane (6 mL x 3). The combined organic layer was dried with Na_2_SO_4_ with cooling, and then filtered. After it was reduced in volume, the residue was purified with silica-gel chromatography with cooling using dichloromethane as eluant to give the product. Enantiomeric excess was determined with HPLC with a Chiralcel OD-H column, hexane:isopropanol 9:1, flow = 1mL/min, 11.0 min (*R*), 12.7 min (*S*). The absolute configuration was compared with the elution order of the known compounds from the literature [22].

## 4. Conclusions

A new pair of biphenyl atropisomeric CF_3_-containing di-alcoholic **(*R***,***S*_a_**,***R*)-2** and **(*R***,***R*_a_**,***R*)-2** and their mirror images were successfully synthesized from key steps including chiral resolution of racemic alcoholic intermediates **b** and homocoupling of the enantiopure **b**. The di-alcohols were expected to be conformationally flexible at the biphenyl backbone but turned out to be conformationally stable to their corresponding *S*_a_ or *R*_a_ configurations. Their favored biphenyl arrangements were shown in X-ray analyses and arose from formation of intramolecular hydrogen bonds from their hydroxyl groups. Their conformational stability was also demonstrated in solution state analysis as no atropisomerization was observed with ^1^H NMR analysis even in polar protic solvents. Since they are resistant to atropisomerization, the atropisomers **2** were separately investigated in a catalytic asymmetric *N*-nitroso aldol reaction and exhibited different enantioselectivities and reactivities. The results presented in this work are significantly governed by the strong electron-withdrawing properties of the CF_3_ groups. To the best of our knowledge, strategic synthesis of stereogenic atropisomeric pairs of organocatalysts with CF_3_-enhanced hydrogen bonding is rarely reported; further works on optimization with various substituent combinations are ongoing.

## Figures and Tables

**Table 1:**
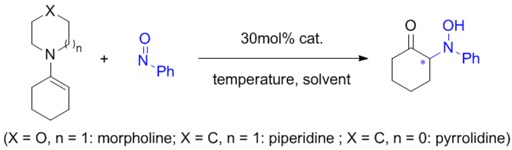
Catalytic asymmetric *N*-nitroso aldol reaction of enamine and nitrosobenzene^a.^

Entry	Catalyst^a^	Solvent	Temperature	Enamine	Yield%^b^	Ee%^c^
1	**1**	Toluene	−50 °C	Morpholine enamine	63	12 (*S*)
2	***(S*,*R*_a_,*S*)-2**	Toluene	−50 °C	Morpholine enamine	38	44 (*S*)
3	***(S*,*S*_a_,*S*)-2**	Toluene	−50 °C	Morpholine enamine	23	8 (*S*)
4	***(S*,*R*_a_,*S*)-2**	Toluene	−50 °C	Piperidine enamine	24	30 (*S*)
5^d^	***(S*,*R*_a_,*S*)-2**	Toluene	−50 °C	Pyrrolidine enamine	-	-
6	***(S*,*R*_a_,*S*)-2**	DCM	−50 °C	Morpholine enamine	55	14 (*S*)
7	***(S*,*R*_a_,*S*)-2**	Et_2_O	−50 °C	Morpholine enamine	62	41 (*S*)
8	***(S*,*R*_a_,*S*)-2**	THF	−50 °C	Morpholine enamine	64	31 (*S*)
9	***(S*,*R*_a_,*S*)-2**	Toluene	−80 °C	Morpholine enamine	64	50 (*S*)
10^e^	***(S*,*R*_a_,*S*)-2**	Toluene	−80 °C	Morpholine enamine	58	46 (*S*)
11^f^	***(S*,*R*_a_,*S*)-2**	Toluene	−80 °C	Morpholine enamine	47	34 (*S*)
12	***(R*,*S*_a_,*R*)-2**	Toluene	−80 °C	Morpholine enamine	61	48 (*R*)
13	***(R*,*R*_a_,*R*)-2**	Toluene	−80 °C	Morpholine enamine	53	20 (*R*)

^a^ Reactions were run with 0.05 mmol of catalyst, 0.17 mmol nitrosobenzene in 0.6 mL solvent at −50 °C or −80 °C under nitrogen. Then 0.17 mmol enamine in 0.33 mL solvent was added over 1 h. The mixture was stirred for 1 day (−50 °C) or 2 days (−80 °C). Then the product was isolated by column chromatography with silica gel with cooling. ^b^ Isolated yields. ^c^ Determined by HPLC with chiral columns Chiralcel OD-H, and the absolute configuration assigned by comparison with known compounds. ^d^ No product was observed. ^e^ 20 mol% of catalyst was used. ^f^ 10 mol% of catalyst was used.

## Supplementary Materials

The following are available online. Figure S1: HPLC chromatograms of **(*S*)-b** and **(*R*)-b**, Figure S2: Circular dichroism spectrum of **(*S*,*R*_a_,*S*)-2** and **(*S*,*S*_a_**,***S*)-2**, Figure S3 and S4: ^1^H NMR and ^13^C NMR of **racemic-b**, Figure S5 and S6: ^1^H NMR and ^13^C NMR of **(*R*)-c**, Figure S7 and S8: ^1^H NMR and ^13^C NMR of **(*S*)-c**, Figure S9 and S10: ^1^H NMR and ^13^C NMR of **chiral-b**, Figure S11 and S12: ^1^H NMR and ^13^C NMR of **(*R***,***S*_a_**,***R*)-2**, Figure S13 and S14: ^1^H NMR and ^13^C NMR of **(*R***,***R*_a_**,***R*)-2**, Table S1: Crystal data and structure refinement of **(*S*)-c**, Table S2: Crystal data and structure refinement of **(*R***,***S*_a_**,***R*)-2** and **(*R***,***R*_a_**,***R*)-2**.
