# Supplementary material for: Synthesis of a Conformationally Stable Atropisomeric Pair of Biphenyl Scaffold Containing Additional Stereogenic Centers
Source: Molecules. 2019 Feb 12;24(3):643. doi: 10.3390/molecules24030643 (PMC6385101; doi:10.3390/molecules24030643)
Supplement: Supplementary file 1 [file molecules-24-00643-s001.pdf]

Synthesis of a Conformationally Stable Atropisomeric Pair of Biphenyl Scaffold Containing Additional Stereogenic Centers

Chi-Tung Yeung [1,2], Wesley Ting Kwok Chan [2], Wai-Sum Lo [1,2], Ga-Lai Law [1,2] and Wing-Tak Wong [1,2,*]

[1] The Hong Kong Polytechnic University Shenzhen Research Institute, Shenzhen 518000, PR China; chitung.yeung@polyu.edu.hk (C.-T.Y.); wai-sum.lo@polyu.edu.hk (W.-S.L.); ga-lai.law@polyu.edu.hk (G.-L.L)

[2] State Key Laboratory of Chemical Biology and Drug Discovery, Department of Applied Biology and Chemical Technology, The Hong Kong Polytechnic University, Hung Hom, Kowloon 999077, Hong Kong; wesley.chan@polyu.edu.hk

* Correspondence: w.t.wong@polyu.edu.hk; Tel.: +852-34008789

Table S1. Crystal data and structure refinement of **(*S*)-c**, [(1*S*,4*R*)-((*S*)-2-(2-bromophenyl)-1,1,1-trifluoropropan-2-yl) 4,7,7-trimethyl-3-oxo-2-oxabicyclo[2.2.1]heptane-1- carboxylate].

Empirical formula	C19 H20 Br F3 O4
Formula weight	449.26
Temperature	295(2) K
Wavelength	0.71073 Å
Crystal system, space group	Orthorhombic, P 21 21 21
Unit cell dimensions	a = 7.6709(4) Å alpha = 90 °
	b = 12.9841(8) Å beta = 90 °
	c = 19.3444(12) Å gamma = 90 °
Volume	1926.7(2) A^3
Z	4
Absorption coefficient	2.183mm^{-1}
F(000)	912
Crystal size	0.26 x 0.26 x 0.10 mm
θ range for data collection	2.626 - 27.510

Limiting indices	-9 ≤ h ≤ 7, -16 ≤ k ≤ 16, -24 ≤ l ≤ 25
Reflections collected / unique	19572
Completeness to θ	99.9%
Absorption correction	Multi-scan
Max. and min. transmission	0.8113 / 0.6007
Refinement method	Full-matrix least-squares treatment on F^2
Data / restraints / parameters	4392 / 0 / 249
Goodness-of-fit on F^2	1.000
Final R indices [I>2sigma(I)]	R1 = 0.0395, wR2 = 0.0787
R indices (all data)	R1 = 0.856, wR2 = 0.0917
Absolute structure parameter	0.021(12)
Largest diff. peak and hole	0.209 and -0.311 eÅ^{-3}

Table S2. Crystal data and structure refinement of **(*R*,*S*$_a$,*R*)-2** and **(*R*,*R*$_a$,*R*)-2**

	**(*R*,*S*$_a$,*R*)-2**	**(*R*,*R*$_a$,*R*)-2**
Empirical formula	C18H16F6O2	3(C18H16F6O2), H2O
Formula weight	378.31	1152.94
Crystal system, space group	Orthorhombic, P212121	Triclinic, P1
Unit cell dimensions	a=11.1649(3)	a=9.1772(21)
	b=11.1810(3)	b=10.7645(24)
	c=13.9971(3)	c=14.8317(33)
	alpha=90	alpha=78.2437(46)
	beta=90	beta=76.8138(49)
	gamma=90	gamma=75.0659(50)
Volume	1747.32	1361.91
Absorption coefficient	1.196 mm^{-1}	1.173 mm^{-1}
F(000)	776	592

Crystal size	0.28 x 0.36 x 0.60	0.26 x 0.44 x 0.54
θ range for data collection	5.063 - 72.303	3.097 - 72.254
Limiting indices	-13 ≤ h ≤ 13	-10 ≤ h ≤ 11
	-13 ≤ k ≤ 13	-13 ≤ k ≤ 13
	-17 ≤ l ≤ 17	-18 ≤ l ≤ 18
Reflections collected / unique	15951 / 3447	49673 / 9984
Completeness to θ	99.9%	98.6%
Absorption correction	Multi-scan	
Max. and min. transmission	0.7306 / 0.5339	0.7501 / 0.5699
Refinement method	Full-matrix least-squares treatment on F^2	
Data / restraints / parameters	3447 / 0 / 241	9984 / 8 / 728
Goodness-of-fit on F^2	1.051	1.057
Final R indices [I>2σ(I)]	R1 = 0.0466	R1 = 0.03535
	wR2 = 0.1402	wR2 = 0.1018
R indices (all data)	R1 = 0.0478	R1 = 0.0518
	wR2 = 0.1421	wR2 = 0.1057
Absolute structure parameter	0.01(3)	0.04(2)
Largest diff. peak and hole	0.148 and -0.218 eÅ^{-3}	0.161 and -0.148 eÅ^{-3}

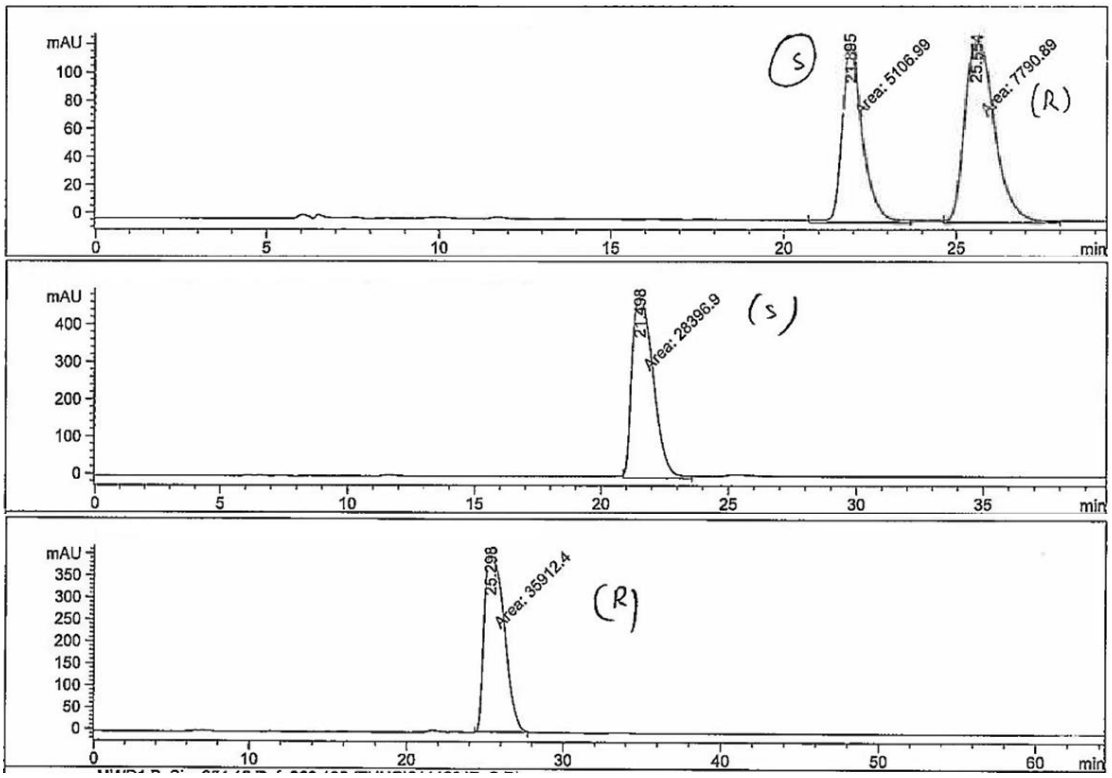

Figure S1. HPLC chromatograms of (top) a mixture of **(*R*)-b** and **(*S*)-b**, (middle) **(*S*)-b** and (bottom) **(*R*)-b**.

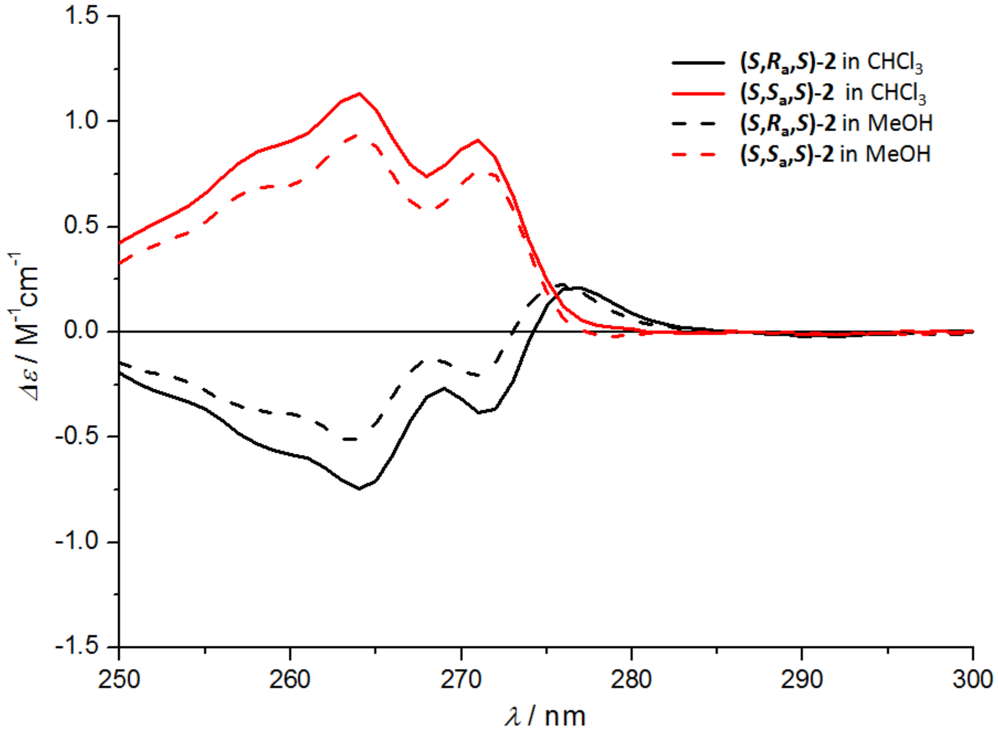

Figure S2. Circular dichroism spectra of **(*S*,*R*a,*S*)-2** and **(*S*,*S*a,*S*)-2** in $CHCl_3$ and MeOH (*c* 0.026 M, path 0.1 cm).

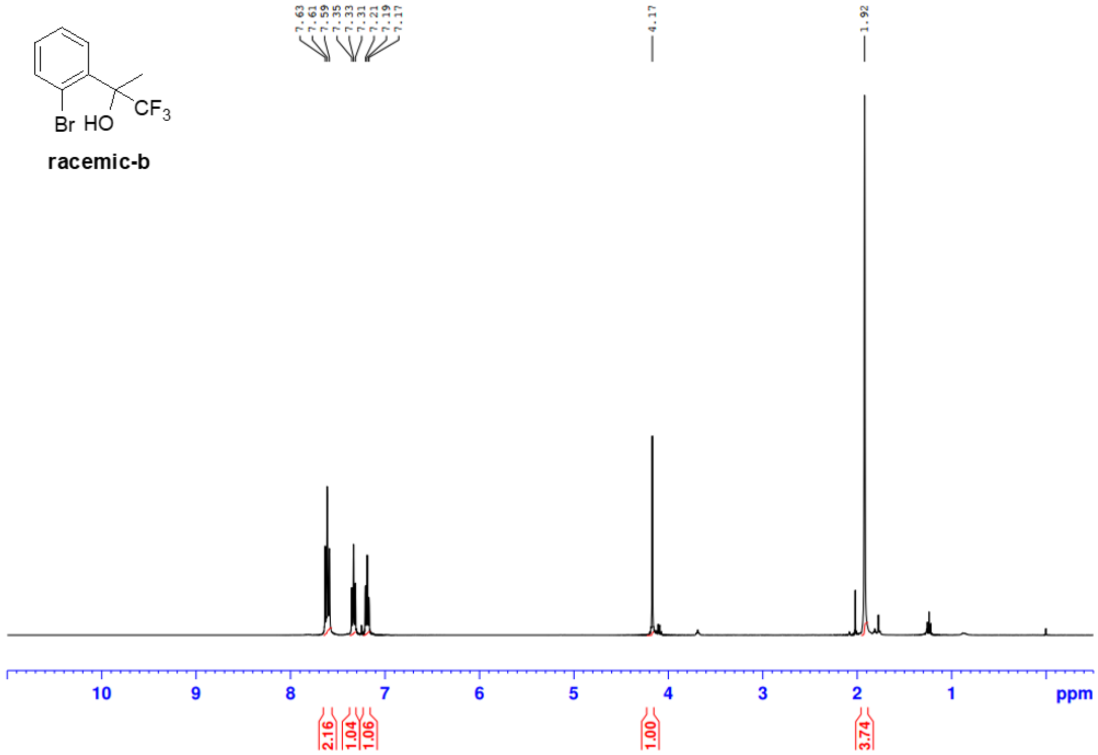

Figure S3. ^{1}H NMR of **racemic-b**.

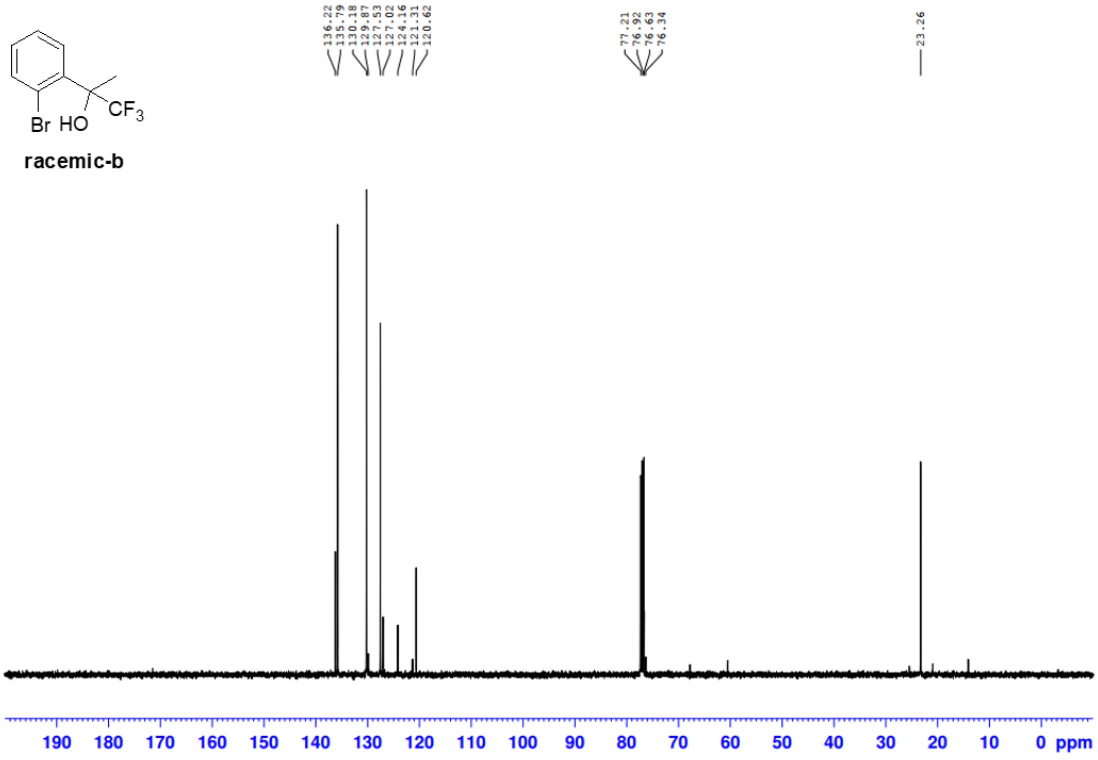

Figure S4. ^{13}C NMR of **racemic-b**.

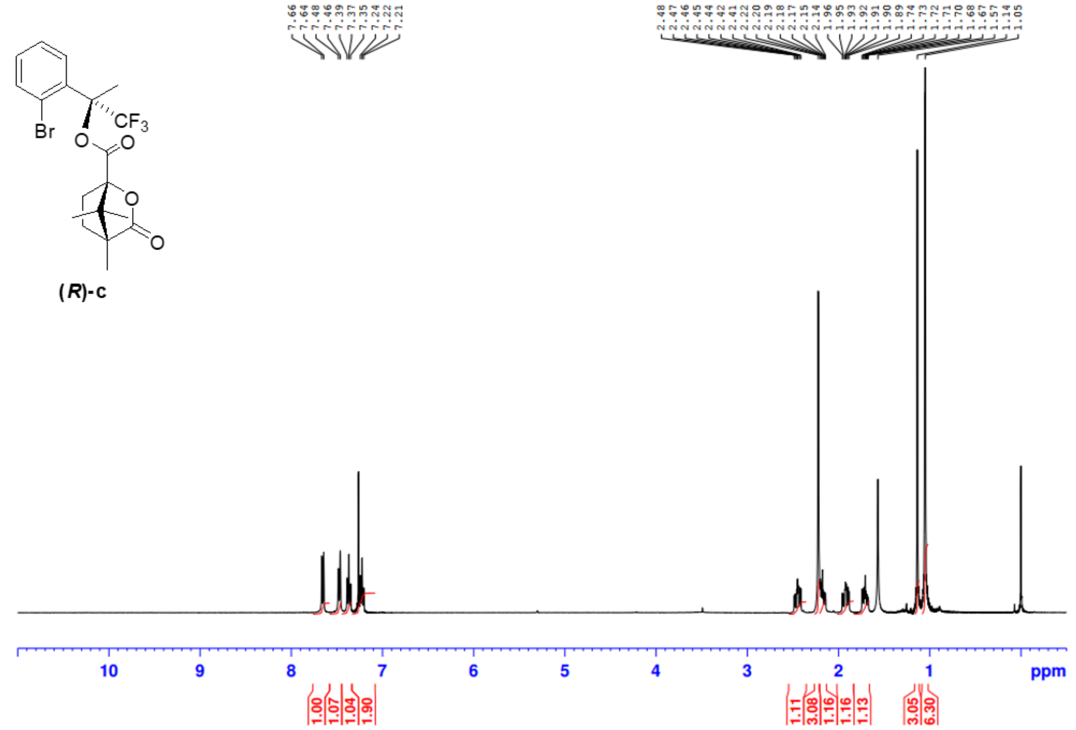

Figure S5. ^{1}H NMR of **(*R*)-c**.

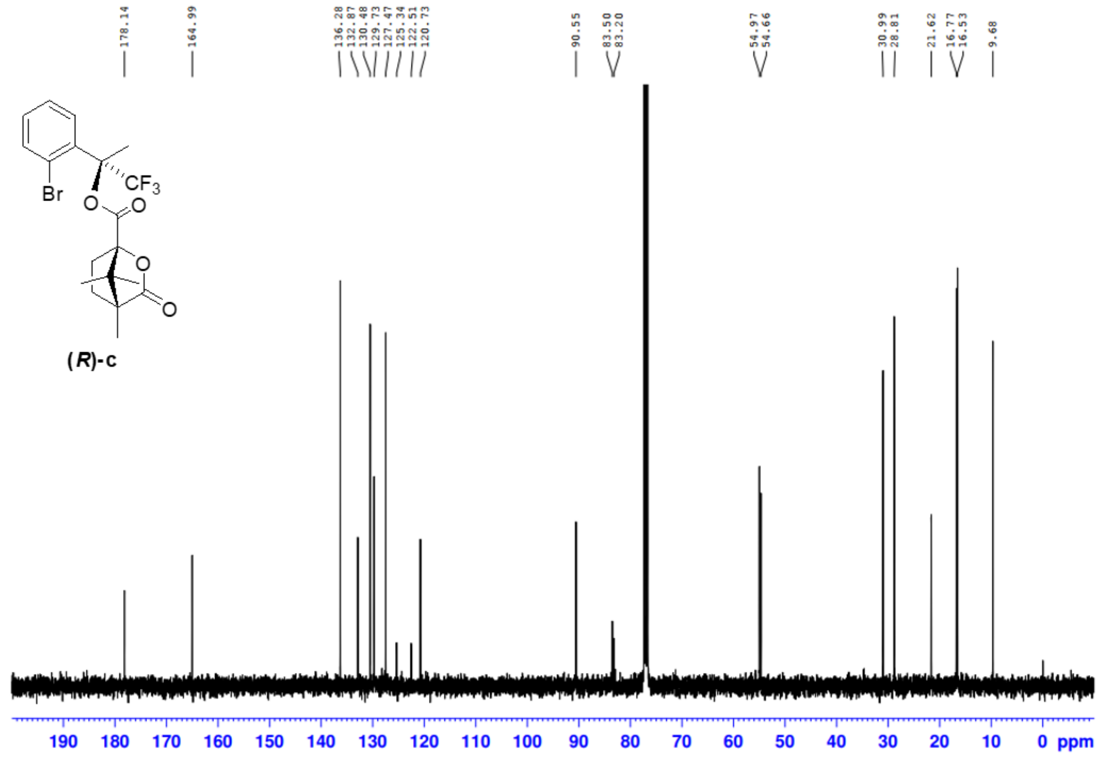

Figure S6. ^{13}C NMR of **(*R*)-c**.

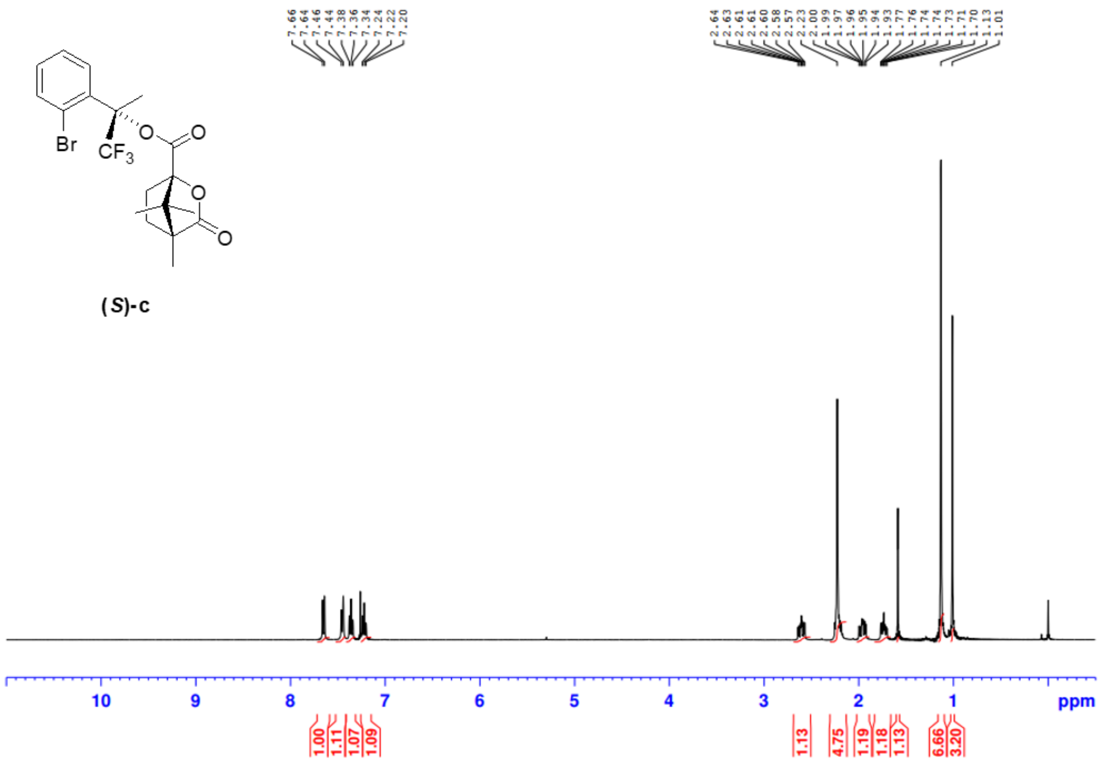

Figure S7. ^{1}H NMR of ***(S)*-c**.

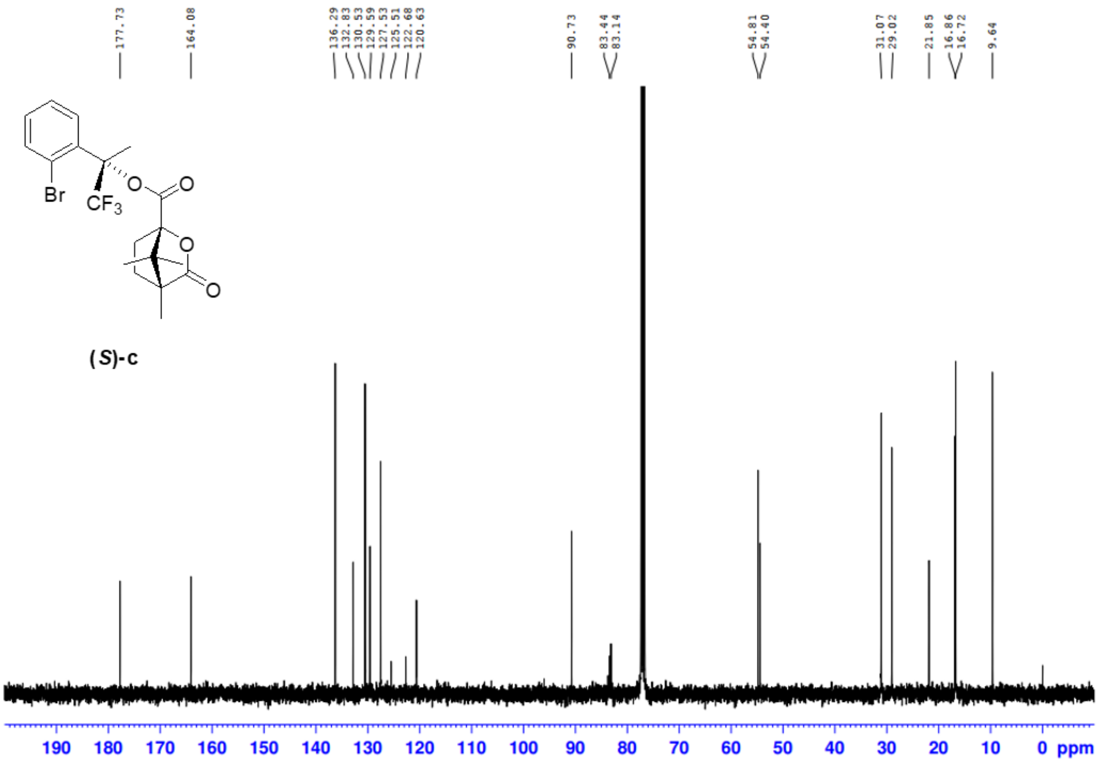

Figure S8. ^{13}C NMR of ***(S)*-c**.

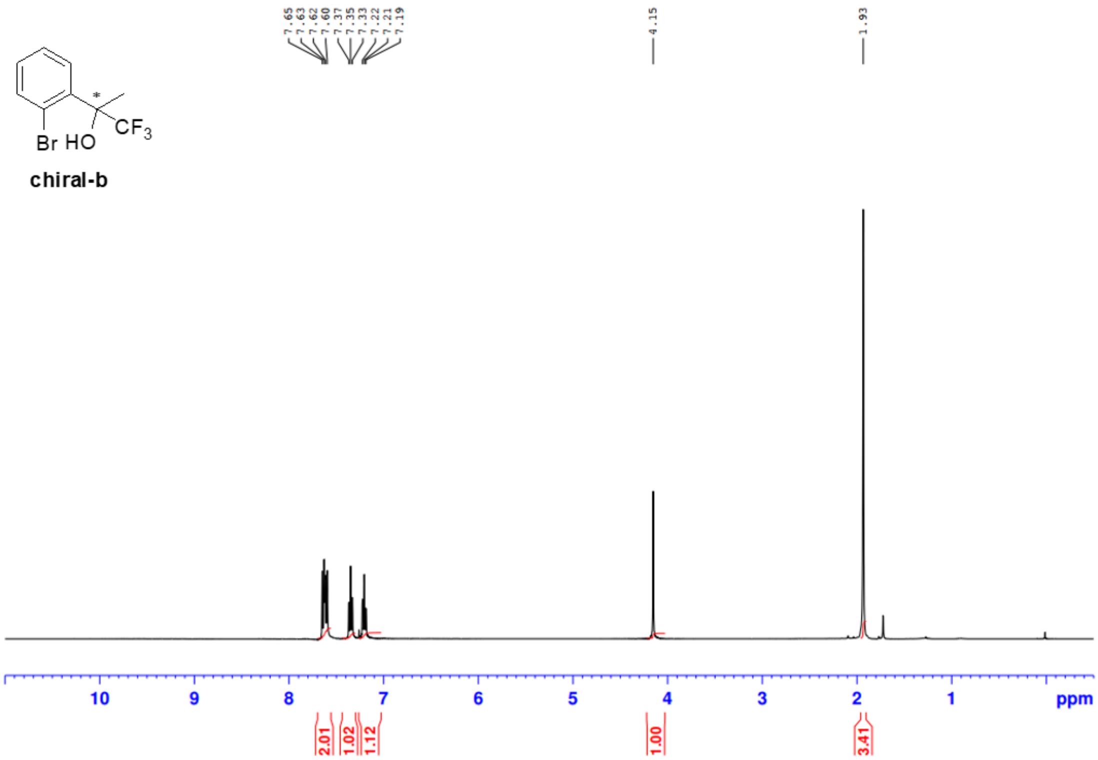

Figure S9. ^{1}H NMR of **chiral-b**.

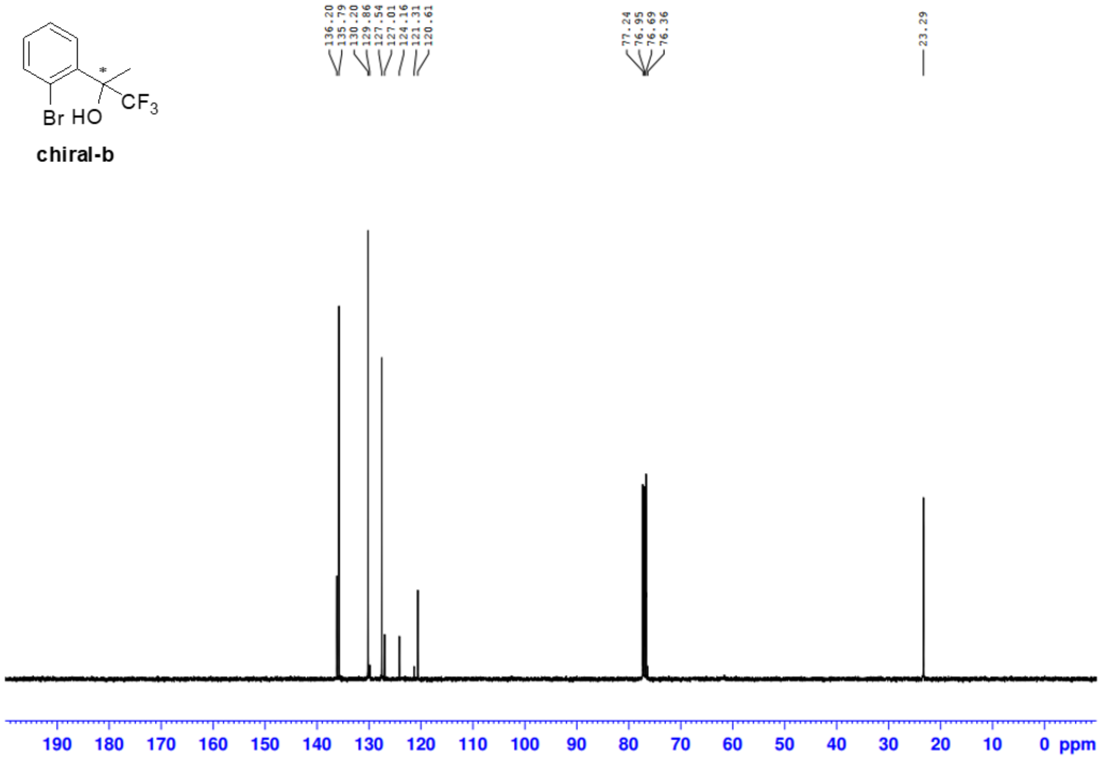

Figure S10. ^{13}C NMR of **chiral-b**.

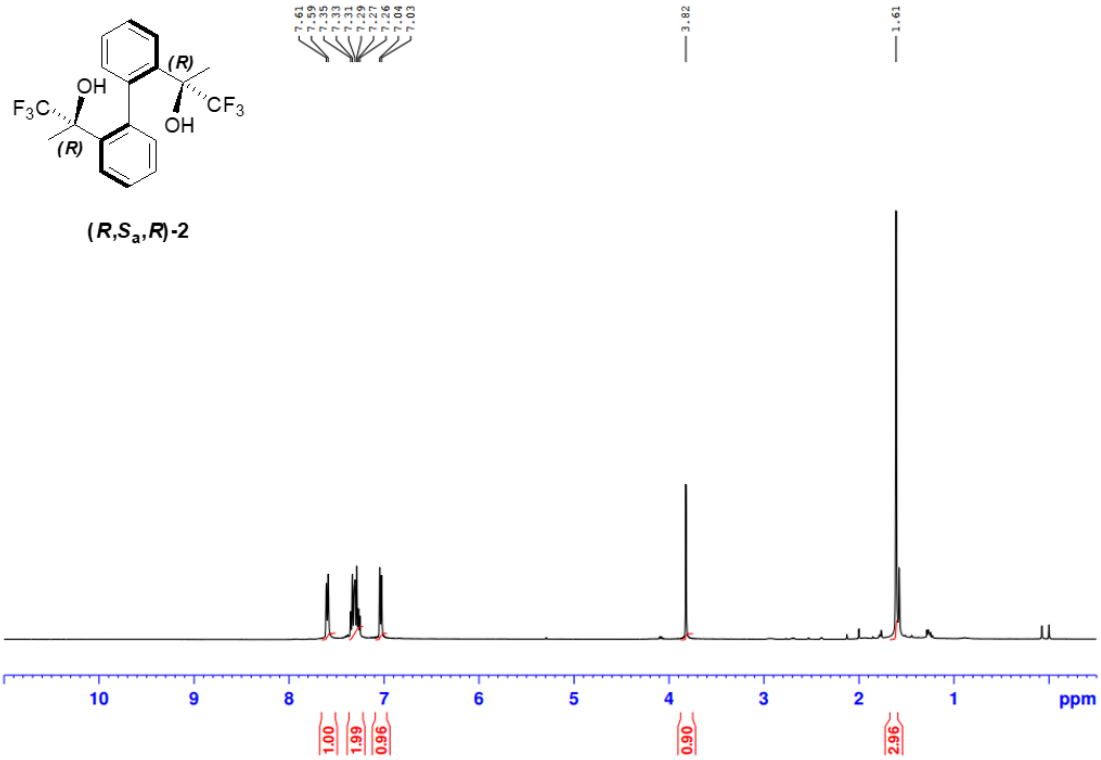

Figure S11. ^{1}H NMR of ***(R*,*S*a,*R*)-2**.

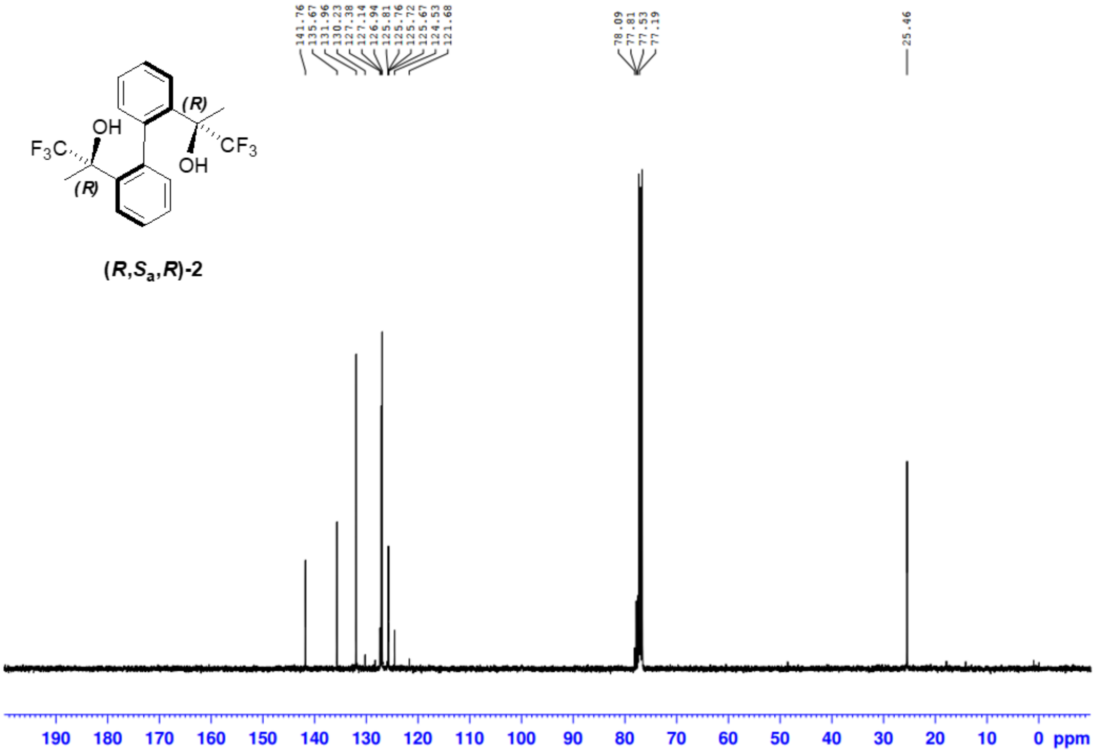

Figure S12. ^{13}C NMR of ***(R*,*S*a,*R*)-2**.

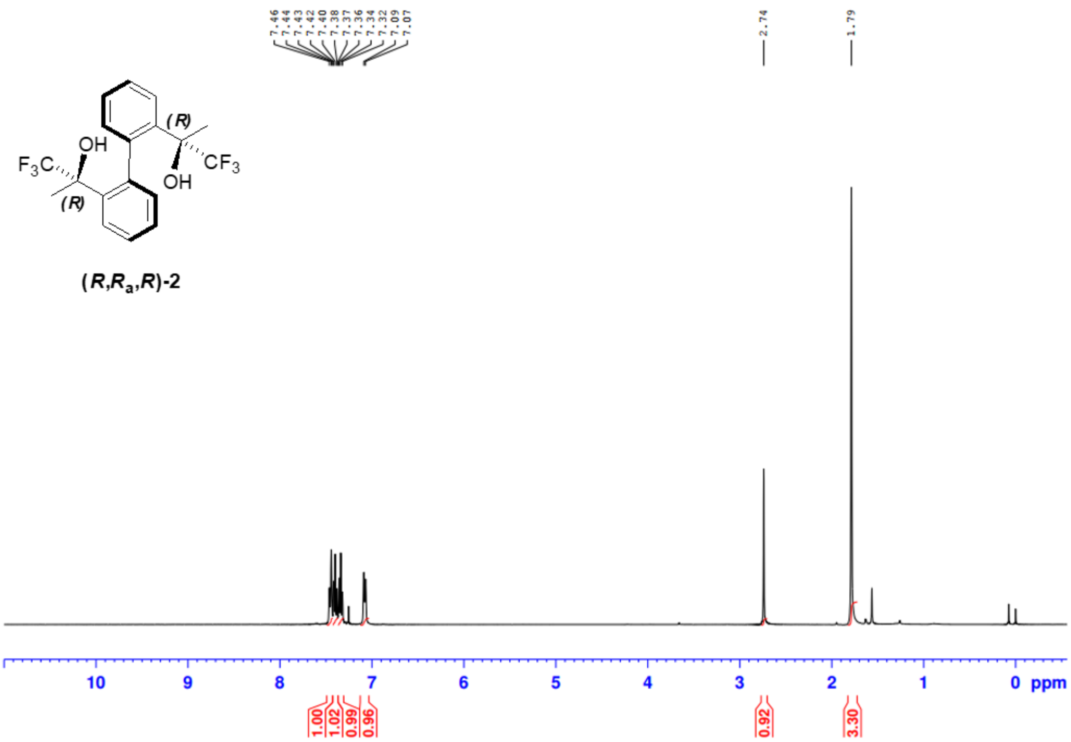

Figure S13. ^{1}H NMR of **(*R*,*R*a,*R*)-2**.

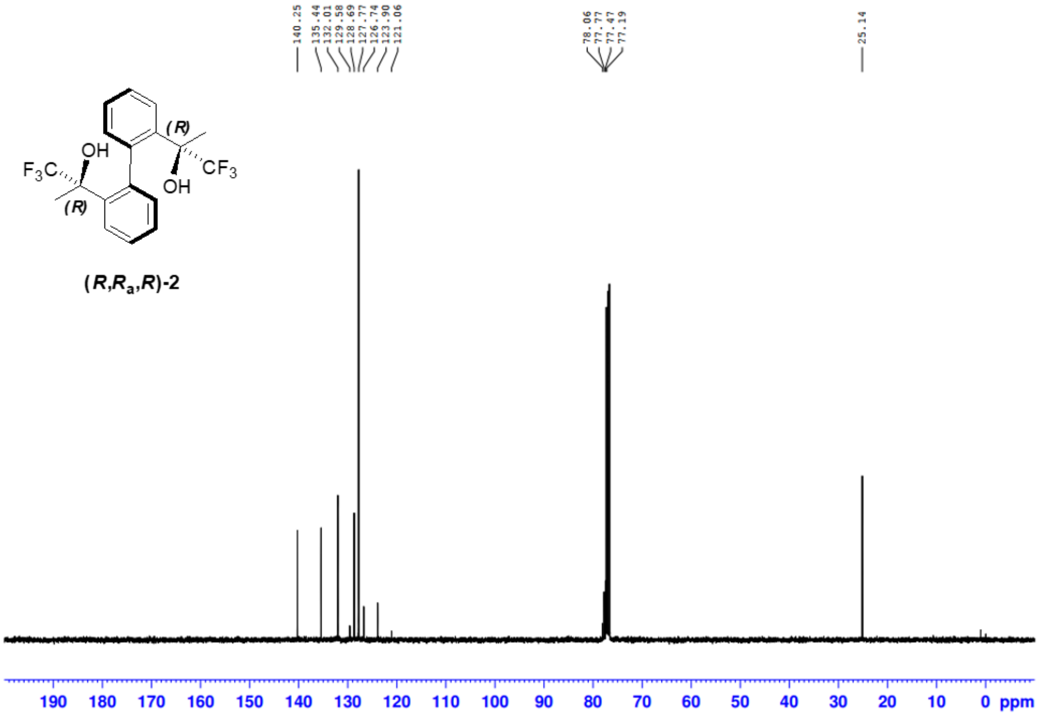

Figure S14. ^{13}C NMR of **(*R*,*R*a,*R*)-2**.
